# *N*-(2′-Hydroxyphenyl)-2-propylpentanamide (OH-VPA), a histone deacetylase inhibitor, induces the release of nuclear HMGB1 and modifies ROS levels in HeLa cells

**DOI:** 10.18632/oncotarget.26077

**Published:** 2018-09-07

**Authors:** Arturo Contis-Montes de Oca, Estefanía Rodarte-Valle, Martha Cecilia Rosales-Hernández, Edgar Abarca-Rojano, Saúl Rojas-Hernández, Manuel Jonathan Fragoso-Vázquez, Jessica Elena Mendieta-Wejebe, Ana María Correa-Basurto, Ismael Vázquez-Moctezuma, José Correa-Basurto

**Affiliations:** ^1^ Laboratorio de Inmunobiología Molecular y Celular, Sección de Estudios de Posgrado e Investigación, Escuela Superior de Medicina, Instituto Politécnico Nacional, Ciudad de México, México; ^2^ Laboratorio de Biofísica y Biocatálisis, Sección de Estudios de Posgrado e Investigación, Escuela Superior de Medicina, Instituto Politécnico Nacional, Ciudad de México, México; ^3^ Laboratorio de Respiración Celular, Sección de Estudios de Posgrado e Investigación, Escuela Superior de Medicina, Instituto Politécnico Nacional, Ciudad de México, México; ^4^ Departamento de Química Orgánica, Escuela Nacional de Ciencias Biológicas, Instituto Politécnico Nacional, Ciudad de México, México; ^5^ Laboratorio de Modelado Molecular y Bioinformática y Diseño de Fármacos, Sección de Estudios de Posgrado e Investigación, Escuela Superior de Medicina, Instituto Politécnico Nacional, Ciudad de México, México

**Keywords:** histone deacetylase inhibitors, high-mobility group box 1 protein, reactive oxygen species, valproic acid derivatives, OH-VPA

## Abstract

N-(2′-Hydroxyphenyl)-2-propylpentanamide (OH-VPA) is a valproic acid (VPA) derivative with improved antiproliferative activity toward breast cancer (MCF-7, MDA-MB-231, and SKBr3) and human cervical cancer cell lines (HeLa) compared to that of VPA. However, the pharmacological mechanism of OH-VPA activity remains unknown. High-mobility group box 1 (HMGB1) is an important enzyme that is highly expressed in tumor cells and has a subcellular localization that is dependent on its acetylation or oxidative state. Therefore, in this study, we analyzed changes in HMGB1 sub-cellular localization and reactive oxygen species (ROS) as well as changes in HeLa cell viability in response to treatment with various concentrations of OH-VPA. This compound is formed by the covalent bond coupling VPA to a phenol group, which is capable of acting as a free radical scavenger due to its chemical similarities to quercetin. Our results show that OH-VPA induces nuclear to cytoplasmic translocation of HMGB1, as demonstrated by confocal microscopy observations and infrared spectra that revealed high quantities of acetylated HMGB1 in HeLa cells. Cells treated with 0.8 mM OH-VA exhibited decreased viability and increased ROS levels compared with the lower OH-VPA concentrations tested. Therefore, the antiproliferative mechanism of OH-VPA may be related to histone deacetylase (HDAC) inhibition, as is the case for VPA, which promotes high HMBG1 acetylation, which alters its subcellular localization. In addition, OH-VPA generates an imbalance in cellular ROS levels due to its biochemical activity.

## INTRODUCTION

The majority of studies that investigate the mechanisms involved in the imbalance of cell growth during tumorigenesis have focused on changes in the structure and function of DNA due to the activation of oncogenes and/or the development of tumor suppressing agents [1]. In this context, high-mobility group box 1 (HMGB1) protein, which has a molecular weight of 24,894 Da, is an important non-histone DNA-binding protein that participates in many cellular processes, including maintaining DNA stability, DNA repair, transcription and recombination. HMGB1 is highly expressed in most tumor types, including cervical cancer and breast cancer [2]. Nuclear HMGB1 can be released into the cytoplasm and then into the extracellular space, where it activates the release of tumor necrosis factor α (TNF-α), interleukin-1 (IL-1) and other inflammatory mediators [3, 4]. The subcellular localization of HMBG1 is dependent upon several posttranslational modifications, such as acetylation, methylation and oxidation [5]. HMGB1 acetylation favors its nuclear-to-cytoplasmic translocation and inhibits its return to the nucleus [2]. The acetylation of HMGB1 on positively charged lysine residues reduces its interaction with chromatin, favoring its translocation into the cytoplasm for uptake by vesicles [6]. HMGB1 is subsequent release from vesicles into the extracellular space where it activates receptors that promote inflammatory signals for immune cells, preserving an inflammatory environment [7].

Histone deacetylases (HDACs) are enzymes that decrease the level of acetylation at exposed lysine residues in histone and non-histone proteins, with HMGB1 being a member of the latter group. Thus, HDAC inhibitors (HDACis) promote HMGB1 acetylation, which induces structural modifications within this protein that alters its subcellular localization. HDACis also exert antiproliferative effects in diverse cancer types through multiple mechanisms [8–10]. Valproic acid (VPA) is a HDACi that has been shown to inhibit the proliferation of various tumors *in vitro* and *in vivo* [11]. However, due to the hepatotoxicity, teratogenicity, and low potency of VPA as an antiproliferative agent, it has not been authorized for use as an anticancer drug [8]. Consequently, Prestegui-Martel *et al*. [12] designed and synthesized a set of VPA derivatives with the goal of identifying molecules with improved antiproliferative activity, yielding a compound named *N*-(2′-hydroxyphenyl)-2-propylpentanamide (OH-VPA). OH-VPA has been reported as an antiproliferative compound with improved potency with respect to VPA [12]. The observed half maximal inhibitory concentration (IC_50_) of OH-VPA in HeLa cells was 0.92 mM, compared to 9.12 mM for VPA, and in breast cancer cells OH-VPA showed better antiproliferative effects than VPA at similar concentrations [12].

Imbalances between the production and elimination of reactive oxygen species (ROS) are known to be involved in carcinogenesis, cell cycle progression, and in promoting a favorable microenvironment for tumor growth and progression [13, 14]. Therefore, the aim of this study was to examine the possible effects of OH-VPA with respect to HMGB1 acetylation and its consequent translocation, as well as to determine its effects on ROS production. To identify a possible mechanism responsible for causing cell death, HeLa cells were treated with OH-VPA at concentrations less than the reported IC_50_ value (0.05, 0.2, 0.4 and 0.8 mM). In this assay, untreated HeLa cells (control) and cells treated with OH-VPA, VPA or lipopolysaccharide (LPS) were evaluated. Since the production of free radicals and energy metabolism need to be finely controlled by tumor cells to ensure their growth and progression, succinate dehydrogenase (SDH) activity (the only enzyme that participates in both the tricarboxylic acid (TCA) cycle and the electron transport chain) was evaluated in all treatment groups using MTT (3-(4,5-dimethylthiazol-2-yl)-2,5-diphenyltetrazolium bromide) assays to assess OH-VPA-induced changes in these mitochondrial processes that are related to free radical production. Confocal microscopy was used to determine whether OH-VPA promotes subcellular translocation of HMGB1 from the nucleus to the cytoplasm, and infrared spectroscopy was used to assess the levels of HMGB1 acetylation in cells treated with OH-VPA or VPA, with a high signal at the 1637 cm^−1^ spectral region belonging to acetyl groups [15]. Finally, ROS production was measured using dichloro-dihydro-fluorescein diacetate (DCFH-DA) assay, and the antioxidant activity of OH-VPA was determined using the DPPH (2,2’-diphenyl-1-picrylhydrazyl) assay to evaluate the free radical scavenging properties of OH-VPA.

## RESULTS

### Effects of VPA and OH-VPA on HeLa cell viability

**Figure 1:**
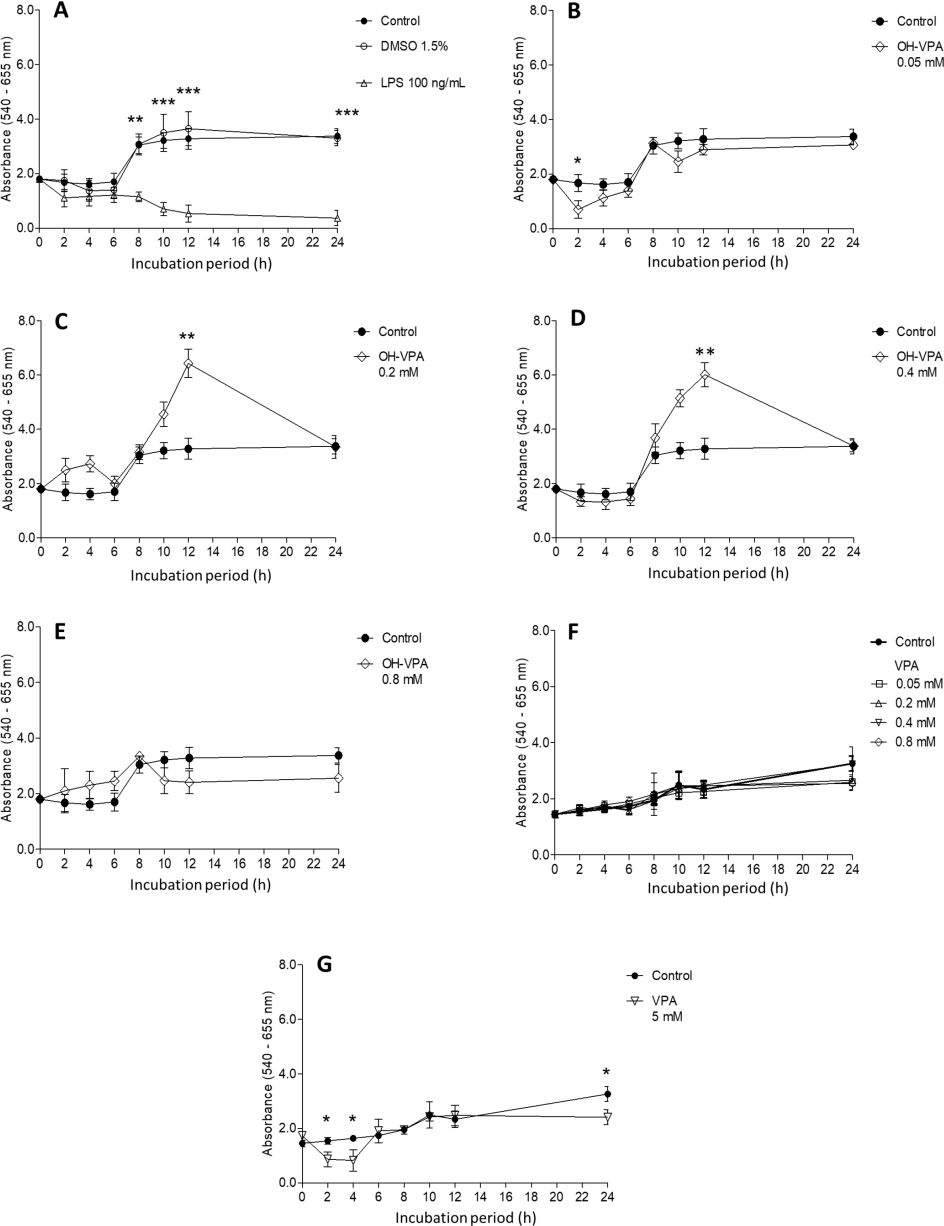
Cell viability evaluation by a MTT assay using HeLa cells incubated for 24 h with various treatments (**A**) No treatment (control), vehicle treatment (DMSO 1.5%) and LPS (100 ng/mL). Treatment with OH-VPA at various concentrations: (**B**) 0.05 mM, (**C**) 0.2 mM, (**D**) 0.4 mM, and (**E**) 0.8 mM. (**F**) Treatment with 0.2, 0.4 and 0.8 mM VPA. (**G**) Upon treating cells with 5 mM VPA, minimal differences were observed at 2, 4 and 24 h, without demonstrating any sustained or important effects. ^*^p < 0.05, ^**^p < 0.01, ^***^p < 0.001.

The SDH activity in HeLa cells was evaluated for cells treated with specific concentrations of LPS, VPA or OH-VPA for set durations of time. Untreated HeLa cells (Figure 1A) exhibited an increase in SDH activity for the first 8 h of incubation, reaching a maximum level that was maintained during the subsequent 24 h. It is possible that in the middle of the cell cycle, HeLa cells undergo a slight enhancement in their metabolic activity due to several factors [16]. The vehicle (DMSO 1.5%) did not produce any significant difference compared to the control (Figure 1A). Treatment with LPS significantly decreased the SDH activity of HeLa cells after 8 h of incubation (Figure 1A). Activation of the signaling pathways that are sensitive to LPS has been shown to diminish the activity of the TCA cycle [17], although the observed effect during the first 8 h was not significant. Treatment of HeLa cells with 0.05 mM of OH-VPA did not elicit changes in SDH activity (Figure 1B). However, treatment of HeLa cells with either 0.2 mM (Figure 1C) or 0.4 mM (Figure 1D) of OH-VPA resulted in an increase in SDH activity in HeLa cells compared to controls at 8 h and 12 h (*p* < 0.05). However, at 24 h, the SDH activity in treated cells was once again indistinguishable from that of the controls. A concentration of 0.8 mM OH-VPA resulted in decreased SDH activity at 6, 10 and 12 h compared to the controls (Figure 1E). Notably, this concentration is close similar to the IC_50_ observed for OH-VPA in HeLa cells (0.92 mM). When these cells were treated with 0.05, 0.2, 0.4 and 0.8 mM VPA, no differences in SDH activity were observed with respect to the controls (Figure 1F). However, treatment of cells with 5 mM VPA resulted in significant differences in SDH activity compared to the controls at 2, 4 and 24 h (Figure 1G), suggesting that VPA diminishes cellular SDH activity at this concentration in a time-dependent manner.

### Evaluation of HMGB1 localization in HeLa cells by confocal microscopy

**Figure 2:**
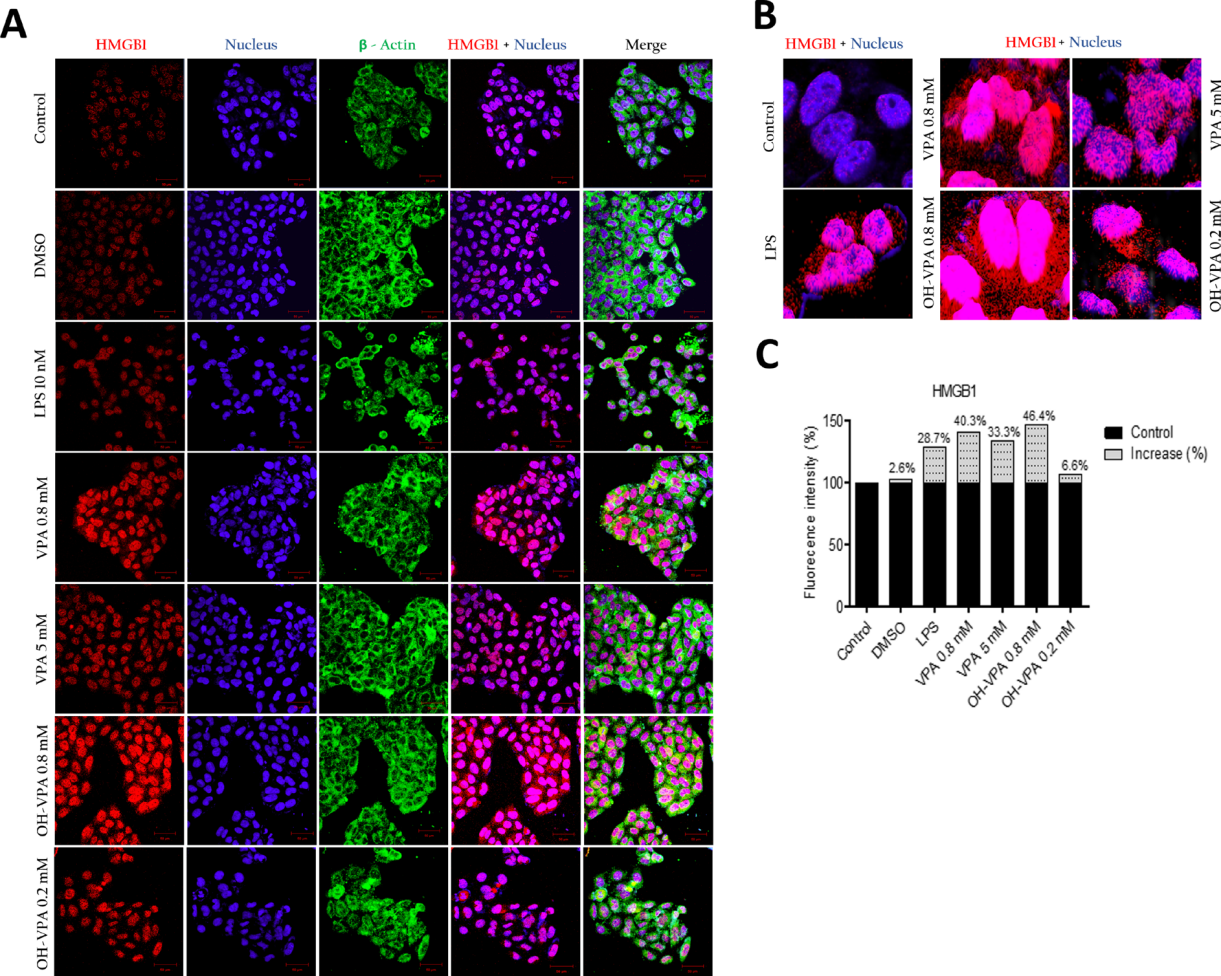
Intracellular location of HMGB1 in HeLa cells at the basal state and in response to treatment with LPS, VPA and OH-VPA (**A**) HMGB1 protein (red) increased in cells that received any of the treatments. HMGB1 translocated into the cytoplasm from the nucleus in all the treated cells, with actin (green) observed in the cytoplasm, and nuclei (blue) delimited to better assess the subcellular localization of HMGB1. (**B**) The percent increase in fluorescence intensity of HMGB1 in HeLa cells pharmacologically treated and stimulated with LPS. (**C**) Merged images showing the increase and visualization of HMGB1 (red) translocated out of the nucleus.

Because we observed decreased SDH activity in HeLa cells treated with 0.8 mM OH-VPA, we evaluated cells treated with this concentration of OH-VPA for 12 h for the localization of HMGB1 via confocal microscopy. As shown in Figure 2A, the fluorescence intensity of HMGB1 (red) increased in cells that received any of the treatments. In addition, we observed translocation of HMGB1 from the nucleus to the cytoplasm in all treated cells, as was shown by the occurrence of positive staining outside of the nuclear borders (Figure 2B). Cells that were treated with 0.2 mM OH-VPA showed a significant increase in the fluorescence intensity (corresponding to HMGB1), 6.6% greater than that of the untreated cells. Interestingly, cells that were treated with 0.8 mM VPA or OH-VPA showed 40.3% and 46.4% increases fluorescence intensity compared to the untreated cells, respectively (Figure 2C).

### Infrared spectra of purified HMGB1 from HeLa cells

**Figure 3:**
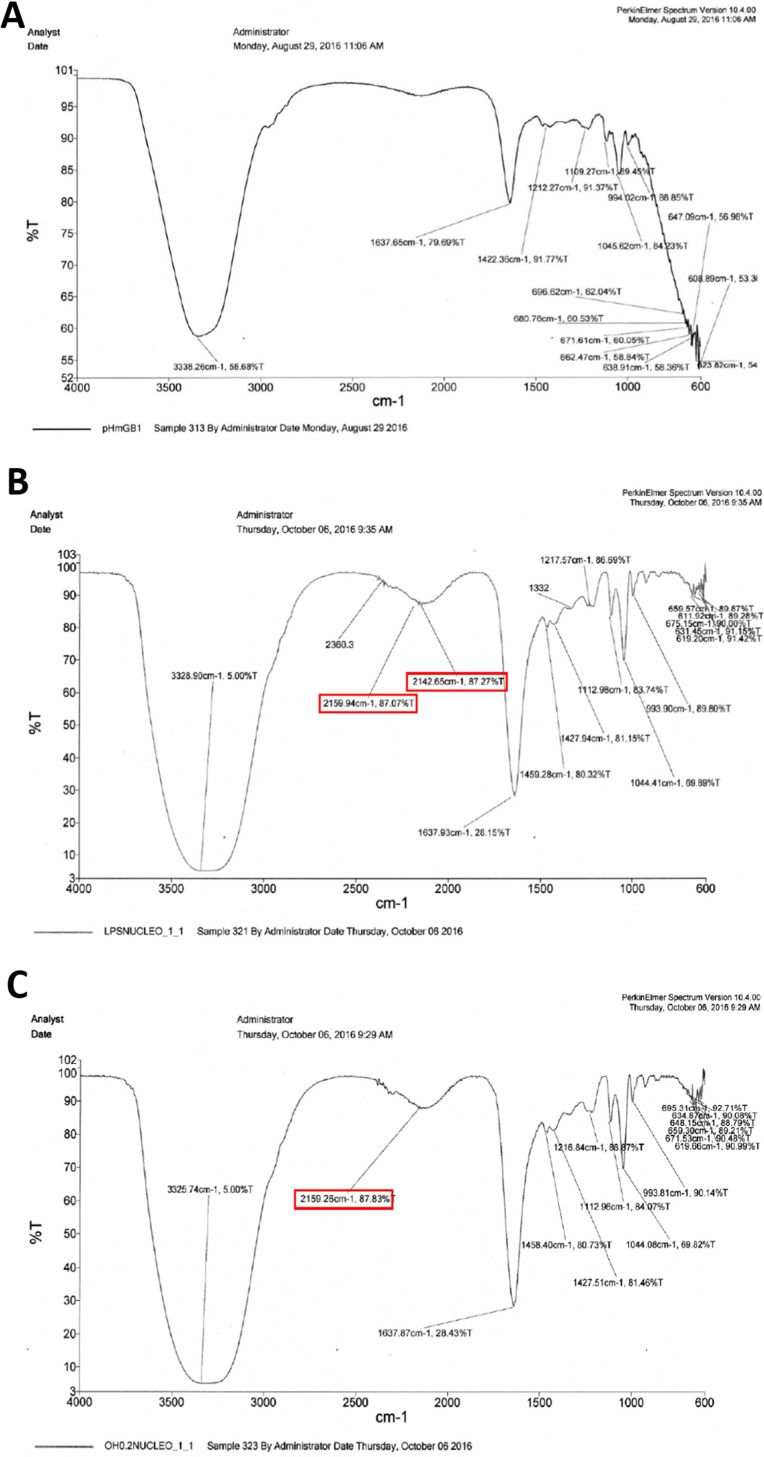
Infrared spectra of HMGB1 purified by immunomagnetic separation from HeLa cells without treatment (**A**), treated with 100 ng/mL LPS for 12 h (**B**), and treated with 0.8 mM OH-VPA for 12 h (**C**). A vibrational modification can be appreciated in the spectrum of the HMGB1 protein from treated cells with LPS and OH-VPA, with change occurring in the 1637 cm^–1^ region, with an increase of 49%.

The infrared spectra of purified HMGB1 (Figure 3) from the control (Figure 3A) and treated (Figure 3B and 3C) HeLa cells demonstrates that the protein purified from cells incubated with LPS or 0.8 mM OH-VPA by immunomagnetic separation had a vibrational change in the region of the amide I band at 1637 cm^−1^, which is within the region reported to correspond to the presence of acetyl groups on proteins [15]. Thus, the treatment of HeLa cells with OH-VPA led to increased acetylation of HMGB1, possibly due to its HDAC inhibitory properties similar to VPA [12, 18].

### Reactive oxygen species (ROS)

**Figure 4:**
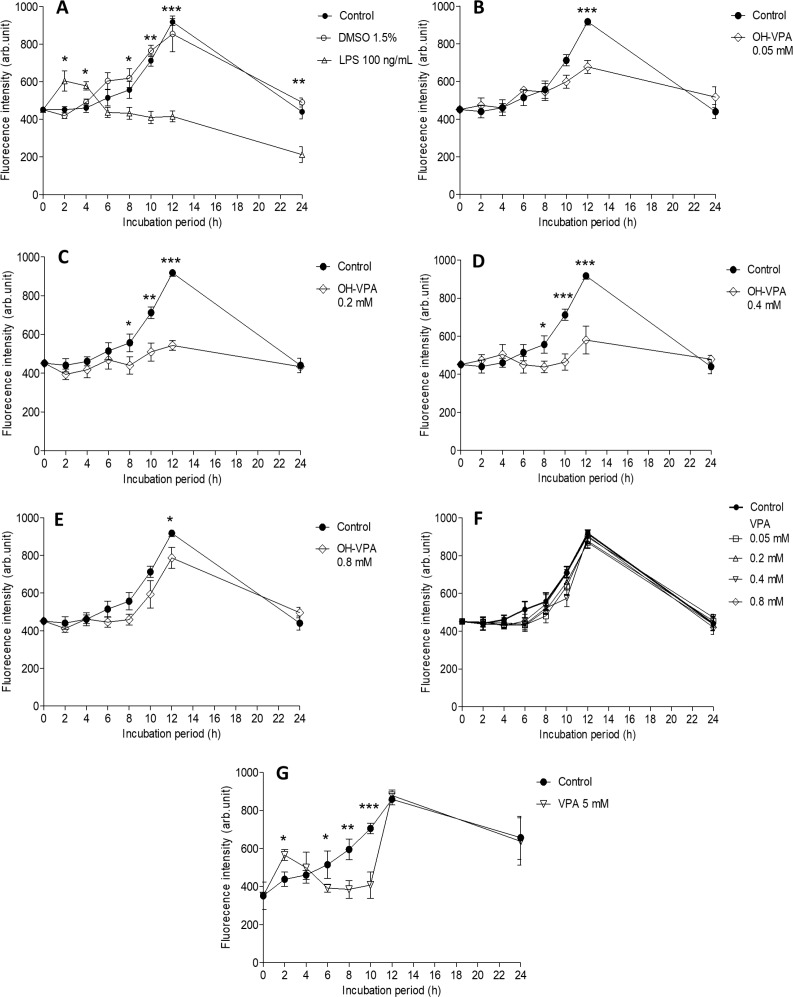
Measurement of reactive oxygen species (ROS) in HeLa cells incubated for 24 h (**A**) Cells without treatment (control), with vehicle (DMSO 1.5%), or with LPS (100 ng/mL). ^*^p < 0.05 control vs LPS. (**B**) Cells treated with OH-VPA at concentrations of 0.05 mM, (**C**) 0.2 mM, (**D**) 0.4 mM, and (**E**) 0.8 mM. (**F**) After 12 h of treatment, a significant decrease in ROS was observed at all concentrations tested and was most notable at 8 h with 0.2 and 0.4 mM OH-VPA. ^*^ *p* < 0.05 control vs OH-VPA. (**G**) Using VPA at 5 mM. ^*^ *p* < 0.05 control vs VPA. ^*^p < 0.05, ^**^p < 0.01, ^***^p < 0.001.

From 6-12 h of the HeLa cell culture period, ROS levels in the untreated cells (control) gradually increase, reaching a maximum value at 12 h (Figure 4A). The increase in ROS levels in cells was likely due to their cell-division cycle [19], and the treatment of cells with vehicle (DMSO 1.5%) did not result in a significant difference compared to the control (Figure 4A). Cells treated with LPS (positive control) showed greater fluorescence intensity during the first 2 h, indicating higher levels of ROS during this period, which was followed by a significant and persistent decrease in this parameter compared to the controls (Figure 4A). Given that the proliferation rate of HeLa cells enhances with increasing levels of ROS, these results correlate with those of the SDH activity assay. Decreased ROS levels were observed upon treating cells with 0.05 mM OH-VPA (Figure 4B), and this effect became more evident after cells were incubated for 12 h with 0.2 mM (Figure 4C) or 0.4 mM OH-VPA(Figure 4D). However, when HeLa cells were treated with 0.8 mM VPA, ROS production was similar to that observed in the control cells (Figure 4E). In addition, when cells were treated with VPA at the same concentrations tested for OH-VPA, a significant difference in ROS production compared to the control group was not observed (Figure 4F). However, when cells were treated with 5 mM VPA (Figure 4G), at which concentration VPA has an antiproliferative effect [11], HeLa cells showed a sharp increase in ROS levels after the first 2 h of incubation, which was followed by a continual decrease in up to 10 h. From 12 to 24 h, ROS levels became similar to those observed for the control group cells treated with 0.8 mM OH-VPA.

### Antioxidant activity of OH-VPA determined by DPPH assay

**Figure 5:**
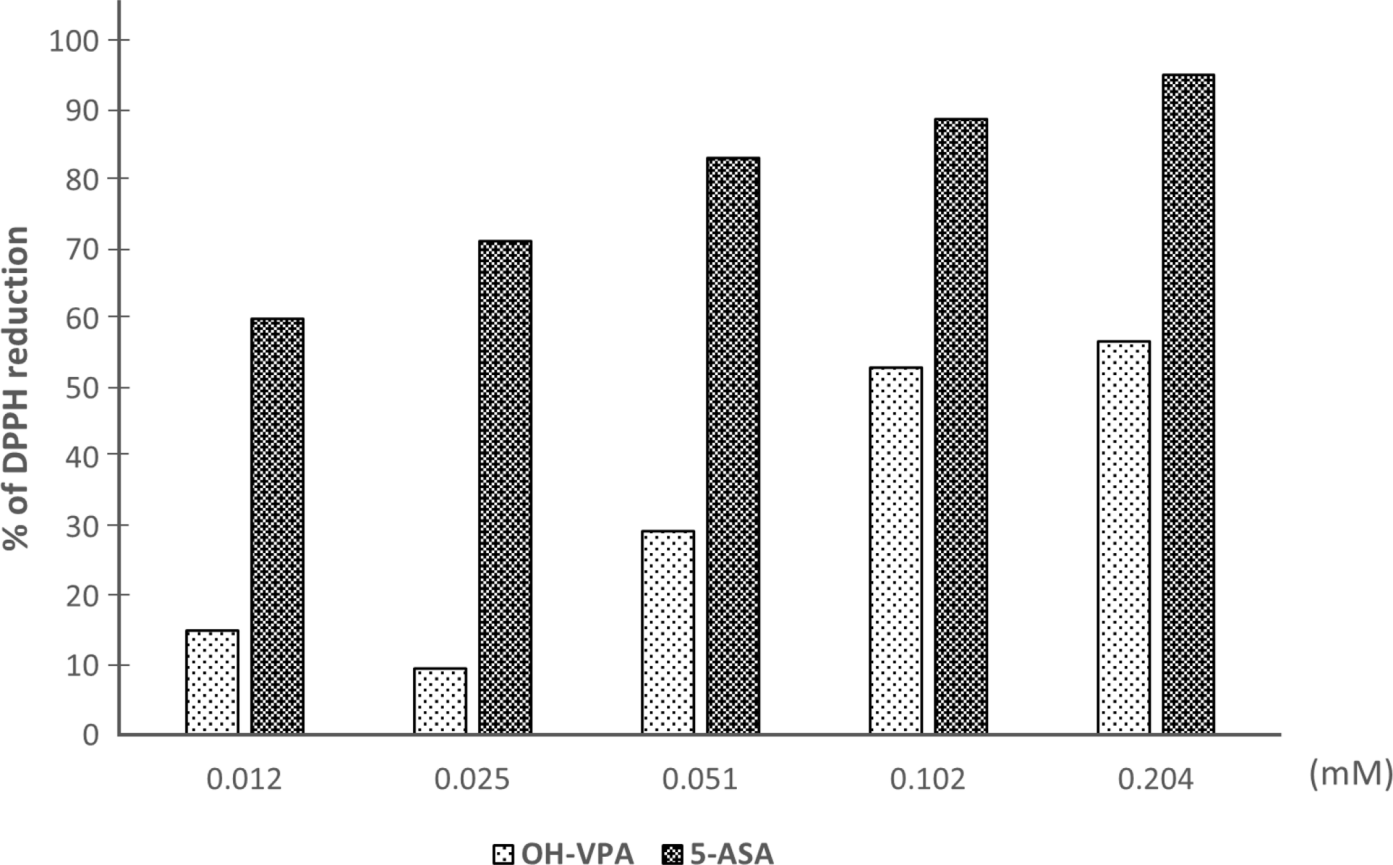
DPPH reduction by OH-VPA and 5-ASA (5-aminosalicylic acid)

OH-VPA has a free radical DPPH reduction activity that is similar tendency to that of 5-aminosalicylic acid (5-ASA), which was used as a reference compound [20, 21]. Figure 5 shows the concentration-dependent reduction in DPPH radicals (%) observed in the presence of 5-ASA or OH-VPA at five different concentrations (0.012, 0.025, 0.051, 0.102, and 0.204 mM, and 0.408 mM). OH-VPA reduced DPPH in a concentration-dependent manner. Compared with 5-ASA, OH-VPA reduced DPPH to a lesser extent at the concentrations assayed, but its concentration-dependent activity ranged from @ 8% at 0.02 mM to @ 60% at 0.24 mM. Thus, these results indicate that OH-VPA has antioxidant activity, probably via free radical trapping due the presence of a phenol group, although this activity was not as strong as that of 5-ASA.

## DISCUSSION

**Scheme 1:**
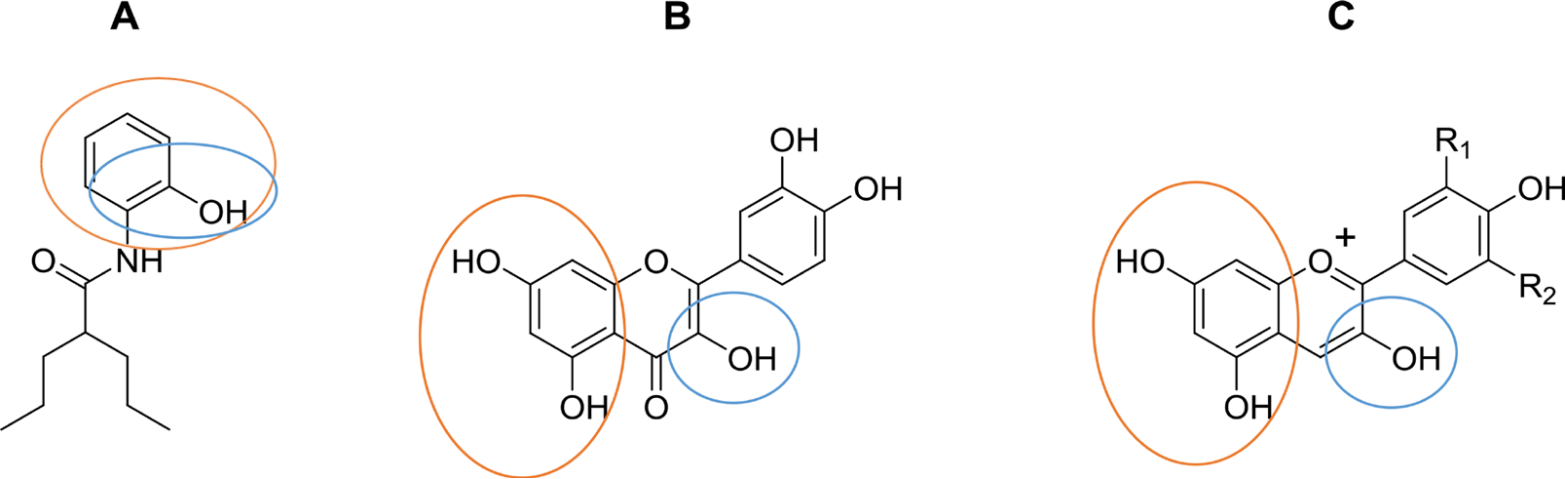
Chemical structures of (**A**) OH-VPA, (**B**) quercetin and (**C**) anthocyanidins. Circles indicate chemical structures able to trap free radical species.

Several investigations of carcinogenesis-related mechanisms have identified important targets for therapy during cancer progression, including changes in redox state, metabolic reprogramming of tumor cells, and the inflammatory environment, among others [22–25]. The HMGB1 protein regulates diverse cell functions, and its overexpression could be a hallmark of cancer, others of which include unlimited replicative potential, angiogenesis, evasion of programmed cell death (apoptosis), self-sufficiency in growth signals, insensitivity to inhibitors of growth, inflammation, tissue invasion and metastasis [7, 26–28]. HMGB1 is modified post-translationally by acetylation and is also sensitive to redox states [5, 6, 29]. In a previous study, *in silico* tools were used to design an aryl derivative of VPA (OH-VPA), a HDACi capable of diminishing the proliferation of HeLa, rhabdomyosarcoma and breast cancer cell lines [12], which could be due to its inhibitory properties on class I HDACs, such as HDAC 1, 3 and 8, which are involved in the deacetylation of HMBG1. VPA, as a HDACi [30], modifies the acetylation state of HMGB1. Thus, the results of the current study showed the effect of OH-VPA on HeLa cells in the context of OH-VPA as a HDACi that modifies the HMGB1 protein and as a free radical scavenger due to its chemical structural similarities to quercetin and anthocyanidins (Scheme 1).

The cell viability assay results showed that HeLa cells treated with 0.2 and 0.4 mM OH-VPA displayed a greater reduction of MTT at 12 h, whereas at 0.8 mM, the opposite effect was observed. In addition, at a concentration of 0.8 mM OH-VPA notably increased nuclear-to-cytoplasmic translocation of HMGB1, similar to that observed when cells were treated with 0.8 mM VPA. In addition, OH-VPA has a phenol moiety, similar to quercetin (Scheme 1), and it has been reported that this compound stimulates the inhibition of HMGB1-induced TNF-α and IL-1β mRNA expression. These observations suggest that quercetin positively influences cell signaling that promotes the expression of proinflammatory cytokines [31]. These results correlate to those obtained in the SDH activity assay, since when HeLa cells were treated with a concentration of OH-VPA close to the IC_50_ [12], the cellular response indicated an inflammatory type. In this scenario, the translocation and accumulation of proinflammatory molecules is increased, such as HMGB1, which is subsequently released into the extracellular medium, which could be due to HDAC inhibitory properties. It has been demonstrated that LPS [32] and VPA [18] cause increased rates of intracellular acetylation of histones. To corroborate the elevated rate of acetylation of HMGB1 in the presence of OH-VPA, we compared the infrared spectra of purified HMGB1 from HeLa cells having undergone treatment with 0.8 mM LPS or OH-VPA. Acetylation of HMGB1 was observed from the high intensity of the absorption peaks in the region of the amide I band at 1637 cm^−1^, among the treatments (HDACi) herein applied to HeLa cells compared to controls. Furthermore, a concentration of 0.8 mM OH-VPA resulted in SDH enzyme activity (similar to the effect produced by LPS) and increased the production of free radicals. In addition, the infrared spectral analysis show that these changes reflect the highest rate of acetylation of HMGB1, which could be due to the inhibitory effect of HDACs by OH-VPA.

ROS concentrations have been evaluated in distinct tumor types, and an association between ROS levels and patient prognosis has been observed [23, 33, 34]. A specific control in the balance of ROS production is characteristic of tumor cells, as ROS is essential for their growth and proliferation [14]. Therefore, the effect observed by OH-VPA on ROS production altered this balance and causes cell death. Our results show that during a 12 h incubation of HeLa cells with 0.2 or 0.04 mM OH-VPA, ROS production decreased relative to control cells, which could be due to OH-VPA acts as a free radical scavenger at these concentrations. However, when OH-VPA was evaluated at 0.8 mM, ROS levels increased, although not more than in control cells. This finding could be explained by observations in previous studies that reported that diverse HDACis, including VPA, can cause sharp increases in ROS production, reaching sufficient levels to induce apoptosis [35–38]. Additionally, OH-VPA is chemically related to quercetin (Scheme 1), which has been reported to increase ROS levels due to its activity on the GSH system and in inducing cell death. As observed from the DPPH experiment, OH-VPA can act as a free radical scavenger due to its shared chemical structure with quercetin. The chemical features that give quercetin the ability to act as an antioxidant are mentioned elsewhere, one of which is a catechol functionality (*ortho*-dihydroxyl) on its B ring, which is also present in OH-VPA (Scheme 1) [39–41].

Therefore, the free radicals produced could be stabilized in the aromatic ring together with the amide bond, producing similar chemical structures with catechol oxidative agents, such as semiquinones and quinones formed due to quercetin altering redox homeostasis [42]. We observed a mechanism in which OH-VPA could act as a free radical scavenger, but the molecules formed as a result of this activity are able to damage antioxidant molecules or enzymes, resulting in an increase in ROS levels and causing cell death [43, 44]. These results are in agreement with previous findings that in response to oxidative stress, HMGB1 is released from the nucleus; therefore, at 0.8 mM of OH-VPA, there is more ROS and increased HMGB1 translocation [7, 22–25]. However, at 0.2 mM OH-VPA, the opposite behavior was observed, with both ROS levels and HMGB1 translocation being decreased at this concentration. This effect could be, due to possible scavenging activity of OH-VPA, which appears the to be dependent on its concentration. This finding allows us to corroborate that OH-VPA acts as an antiproliferative compound at lower concentrations than VPA because at 0.8 mM, this compound produced effects that were only observed at 5 mM VPA. In addition, OH-VPA and VPA could share an antiproliferative mechanism because both molecules have VPA structure, and the improved activity of OH-VPA could be related to its antioxidant properties.

**Figure 6:**
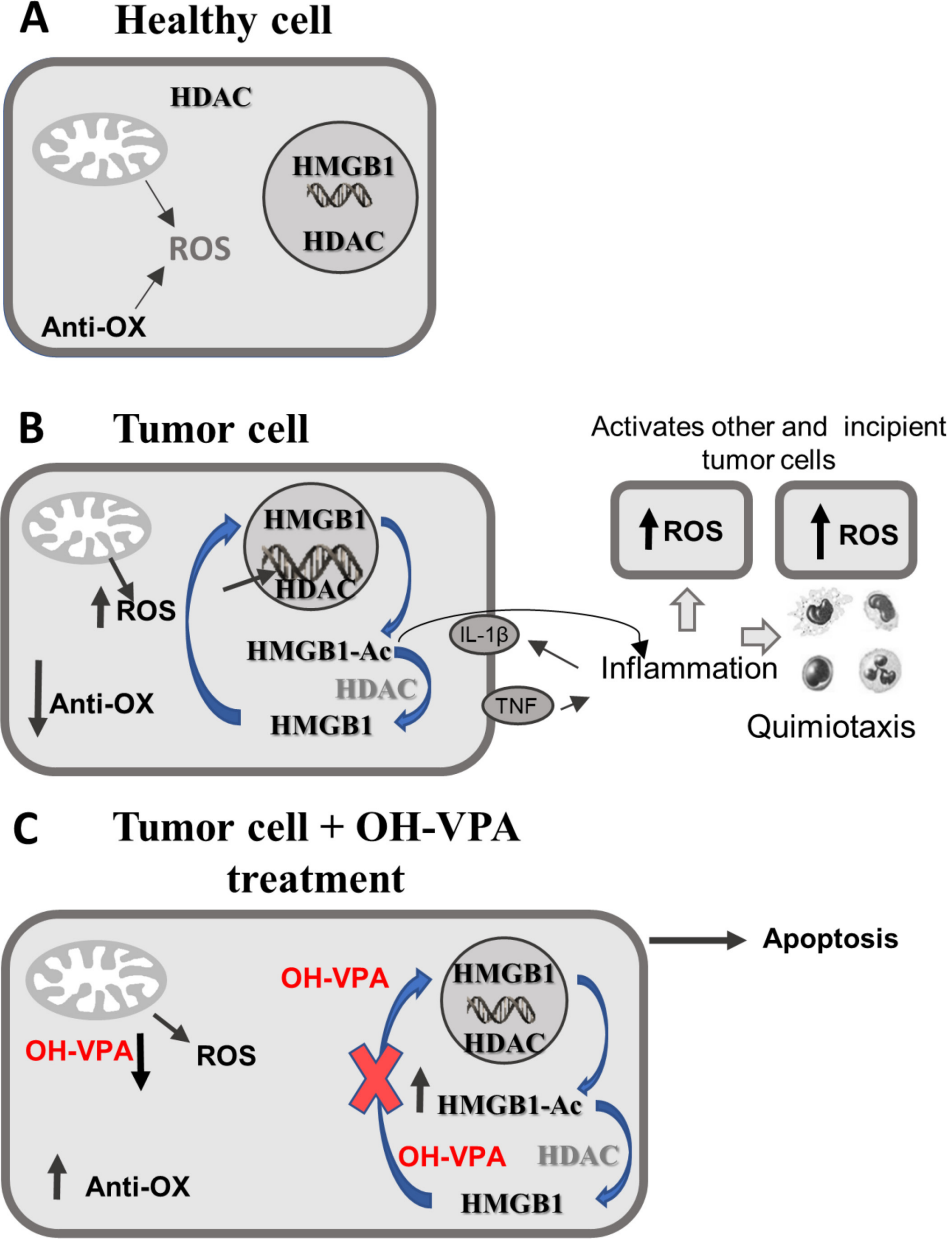
Modifications in ROS production, as well as in HDAC inhibition and rate of intracellular HMGB1 acetylation

Finally, Figure 6 depicts a scheme that could partly explain the pharmacological mechanism of OH-VPA. Figure 6A shows healthy cells that possess a controlled metabolism that is dependent on mitochondrial energy production in the form of ATP. Mitochondrial metabolism is accompanied by the production of ROS, which is regulated by the presence of antioxidant mechanisms responsible for maintaining a balance between ROS production and elimination. In turn, the HMGB1 protein is primarily located in the nucleus because the activity of HDAC enzymes favors its deacetylation, ensuring its maintenance within the nucleus due to the negative charges on DNA. However, translocation of HMGB1 to the cytoplasm, as well as its release to the extracellular medium, only occurs when cells receive a stimulus that increases the rate of intracellular acetylation. Figure 6B shows tumor cells with a reprogrammed metabolism that requires increased production of ROS to activate inflammatory promoters, such as IL-1β and TNF-α, which are responsible for activating other tumor or incipient cells and for attracting immune system cells that are capable of preserving a proinflammatory state. In addition, tumor metabolism is characterized by an elevation in the rate of intracellular acetylation of HMGB1 by post-translational modification. This action facilitates its cytoplasmic translocation and subsequent release, and once receiving the appropriate inflammatory stimulus, it acts as another promotor of inflammation. Figure 6C depicts HeLa cells treated with OH-VPA experiencing a remarkable decrease in ROS production, an increase in mitochondrial reductive capacity and a decrease in HMGB1 expression and translocation compared to cells receiving VPA treatment. The rate of acetylation and cytoplasmic translocation of HMGB1 may depend on the inhibitory effect of OH-VPA on HDAC activity. However, since the amount of intracellular HMGB1 is lower, it is estimated that the proinflammatory potential of the treated tumor cells is diminished in the same way. Thus, significant changes in the metabolic activity of tumor cells could condition or favor the activation of apoptotic mechanisms induced by OH-VPA.

## MATERIALS AND METHODS

### Cell culture

HeLa cells (ATCC CCL-2) were cultured in Dulbecco's Modified Eagle medium (DMEM Gibco, Life Technologies, Invitrogen, USA) supplemented with 10% de-complemented fetal bovine serum (FBS, Biowest, Kansas City, MO, USA), as well as with antibiotic and antimycotic agents (Gibco: Thermofisher, product: 15240062) (DMEMc medium). Cultures were maintained at 37°C in a humidified atmosphere with 5% CO_2_. For experiments, cells were seeded into 96-well plates to form cell monolayers. Prior to each experiment, cells were incubated for 3 h in 100 μL of DMEM with 3% FBS to synchronize the cell cycles.

### Determination of cell viability by MTT assays

After seeding 15 × 10^3^ cells per well in transparent 96-well plates, cells were incubated for 24 h in DMEMc medium. Subsequently, the cells were incubated for 12 h with 25 μL of dimethyl sulfoxide (DMSO 1.5%); 0.05, 0.2, 0.4 or 0.8 mM OH-VPA; or with VPA at the same concentrations, as well as at 5 mM because this concentration has been shown to induce a clear antiproliferative effect [45]. In addition, LPS (100 ng/mL) was administered as an additional control since it has been reported to generate free radicals [46, 47]. After incubation, the cell supernatant was removed. Next, DMEMc without phenol red medium with MTT solution (5 mg/mL) was added, after which the cells were incubated at 37°C for 4 h. Subsequently, the MTT-containing medium was removed, formazan salts were solubilized in DMSO, and the absorbance of each well was read at 540–695 nm with a spectrophotometer (Multiskan EX Microplate Photometer, Thermo Scientific, Mexico). All assays were carried out in triplicate [48].

### Localization of HMGB1 by confocal microscopy

To assess the localization of HMGB1 in cells, 20 × 10^3^ cells per well were added to 24-well plates and incubated for 24 h in DMEMc. After cell synchronization, cells were washed with phosphate buffered saline (PBS). The different treatment and control groups included cells treated with OH-VPA (0.2 and 0.8 mM), VPA (0.8 and 5 mM), vehicle (DMSO 1.5%) and LPS (100 ng/mL), which were incubated for 12 h and stimulated with LPS for 3 h. After incubation, cells were fixed with paraformaldehyde (PFA 2%) at 37°C for 20 min and permeabilized (PBS Tween-Triton 0.5%). Subsequently, cells were washed and primary antibodies, including anti-HMGB1 (GTX101277, GeneTex, USA) and anti-β Actin (ab8229, Abcam, USA), were added at a ratio of 1:1000 and incubated overnight. Next, the secondary antibodies, including Donkey anti-Rabbit IgG H&L (Alexa Fluor^®^ 647) and Donkey anti-Goat IgG H&L (Alexa Fluor^®^ 488), were added at a ratio of 1:1000 and the cells were incubated at 4°C for 2 h. Finally, slides were washed in PBS and mounted with VECTASHIELD Mounting Medium with DAPI (Vector Laboratories, Inc. Burlingame, CA, USA). Images were collected and analyzed with an Axioskop 2 mot plus confocal fluorescence microscope (Carl Zeiss, Mexico) at EC Plan-Neofluar 20×/0.5 ph2: LP 650, BP 420-480, LP 505.

### Obtaining cellular fractions enriched with cytoplasmic and nuclear components

Once cells reached a confluence of 9 × 10^6^ in a 75-cm^2^ culture flask, synchronization and subsequent trypsinization was carried out. First, cells were washed with PBS, after which trypsin (10%) was added and the cells were incubated for 3-5 min. Next, DMEMc medium was added and the cell suspension was centrifuged at 600 × g at 4°C for 10 min to obtain the cell pellet, which was resuspended in 2 mL of DMEMc medium to carry out automated cell counting (TC 10, Bio-Rad, Mexico). The cell pellets were washed with PBS and suspended in 1 mL of cold lysis buffer 1 (10 mM Tris HCl, 10 mM NaCl, 15 mM MgCl_2_, 250 mM sucrose, 0.5% NP-40 and 0.1 mM EGTA, pH 7.5) plus 200 μL of the protease inhibitor hydroxymercury benzoic acid (Cat. No.: 55540, Sigma-Aldrich, Mexico). Next, samples were vortexed for 15 s and incubated on ice for 10 min, which was followed by the addition of 4 mL of lysis buffer 2 (30% sucrose, 10 mM Tris HCl, 10 mM NaCl and 3 mM MgCl_2_, pH 7.5). Solutions were centrifuged at 1300 × g at 4°C for 10 min, after which the supernatants were collected, which corresponded to the first cytoplasmic fraction (C1). The remaining pellet was suspended in 1 mL lysis buffer 3 (10 mM Tris HCl and 10 mM NaCl, pH 7.5) plus protease inhibitor, and the solution was centrifuged at 1300 × g at 4°C for 10 min. The supernatant was collected, which corresponded to the second cytoplasmic fraction (C2). Afterwards, the remaining cellular pellet, corresponding to the nuclei, was suspended in the lysis buffer 4 (50 mM HEPES, 420 mM NaCl and 10% glycerol, pH 7.5) and vortexed for 15 s, and then was alternately shaken and incubated on ice for a total of 40 min. The suspension was then centrifuged at 10,000 × g at 4°C for 10 min, to obtain the nuclear extract (NE) in the supernatant, which was collected in another tube where containing protease inhibitor.

### Purification of HMGB1 with magnetic pearls

Enriched cellular fractions (NE, C1 and C2) were used for purification and analysis of the levels of HMGB1 protein. The NE was utilized as the control group, since the localization of HMGB1 in the basal state is predominantly nuclear [5], and cytoplasmic fractions were utilized for cells receiving treatment. Immunomagnetic separation assays were conducted with a kit of magnetic pearls (Sure Beads, Cat. No.: 1614833, Bio-Rad, Mexico), according to the manufacturer's instructions for purification. The final eluate containing purified HMGB1 protein was stored at −70°C.

### Infrared spectrum characterization of purified HMGB1 from HeLa cells

Samples of purified HMGB1 obtained from nuclei and cytoplasm of HeLa cells (treated and control) were subjected to an infrared spectrometry analysis (FT-IR Frontier, Perkin Elmer, USA) by placing 1-5 μL of purified HMGB1 on the surface of the plate reader. The infrared spectrum of the control sample was obtained separately for each component of the solution, which included Laemmli buffer, bidistilled H_2_O and purified HMGB1 protein.

### Measurement of reactive oxygen species (ROS)

In 96-well plates, 15 × 10^3^ cells per well were seeded and allowed to incubate for 24 h in DMEM with 10% FBS. After cell synchronization, the cells were washed with PBS and incubated for 20 min with 75 μL of 20 μM DCFH-DA (Cat. No.:35845, Sigma-Aldrich, Mexico) in DMEM at 37°C.

Subsequently, cells were incubated using the same treatments employed for the MTT assays (see above). Finally, media was removed from all wells and the cells were washed with PBS, leaving 100 μL of RPMI plus 10% FBS in each well for fluorescence spectroscopy measurements with an excitation at 495 nm, an emission at 520 nm, a filter selection of 515 and an entrance slit of 10 mm (LS 55, Perkin Elmer, USA).

### Antioxidant activity of OH-VPA determined by a 2,2’-diphenyl-1-picrylhydrazyl (DPPH) assay

The DPPH assay was carried out as previously described [20, 21]. Briefly, 1 mL of different solutions of 5-ASA (0.408, 0.204, 0.102, 0.051, 0.025 and 0.012 mM), which was dissolved in ethanol, was mixed with 1 mL of 3 × 10^-5^ M DPPH (Cat. No.: D9132, Sigma-Aldrich, Mexico) dissolved in DMSO at room temperature in total darkness. After a 60 min incubation, the absorbance was measured at 517 nm in a UV-Vis Lambda 25 spectrophotometer (PerkinElmer, USA). The scavenging of DPPH radicals by OH-VPA was evaluated in an equimolar ratio to 5-ASA, and the antioxidant activity was compared with the control sample (1 mL DPPH solution + 1 mL compound solution). DMSO was used as a blank, and the antioxidant activity was determined using the following equation: (1-(Ai-Aj)/Ac)*100, where Ai = tested compound solution + DPPH solution, Aj = tested compound solution + DMSO, and Ac = DPPH solution + DMSO.

### Statistical analysis

To determine significant differences when comparing each group with respect to its control, one-way ANOVA and Tukey's stock pair comparison tests were used in the GraphPad Prism 5 statistical package. Data were expressed as the means ± standard deviation (SD). All assays were performed in triplicate, and significance was considered at *p* < 0.05*, p* < 0.01 *or p* < 0.001.

## CONCLUSIONS

OH-VPA exhibits a superior antiproliferative activity than VPA. This activity is associated with modifications in the acetylation of HMGB1, which promotes its translocation from the nucleus to the cytoplasm, as occurs with VPA, indicating that it may also act as a HDAC inhibitor. Furthermore, OH-VPA produces a ROS imbalance due to its ability to act as antioxidant, since it reduced DPPH radicals and shares a chemical structure with known antioxidants, such as quercetin, possibly promoting cancer cell apoptosis. Therefore, this study shows that OH-VPA could be a better anticarcinogenic than VPA by affecting several cell signaling mechanisms. However, more studies should be conducted to evaluate the specific mechanism of HDAC inhibition by OH-VPA.
