# Correction: *N*-(2´-Hydroxyphenyl)-2-propylpentanamide (OH-VPA), a histone deacetylase inhibitor, induces the release of nuclear HMGB1 and modifies ROS levels in HeLa cells

**DOI:** 10.18632/oncotarget.26672

**Published:** 2019-02-05

**Authors:** Arturo Contis-Montes de Oca, Estefanía Rodarte-Valle, Martha Cecilia Rosales-Hernández, Edgar Abarca-Rojano, Saúl Rojas-Hernández, Manuel Jonathan Fragoso-Vázquez, Jessica Elena Mendieta-Wejebe, Ana María Correa-Basurto, Ismael Vázquez-Moctezuma, José Correa-Basurto

**Affiliations:** ^1^ Laboratorio de Inmunobiología Molecular y Celular, Sección de Estudios de Posgrado e Investigación, Escuela Superior de Medicina, Instituto Politécnico Nacional, Ciudad de México, México; ^2^ Laboratorio de Biofísica y Biocatálisis, Sección de Estudios de Posgrado e Investigación, Escuela Superior de Medicina, Instituto Politécnico Nacional, Ciudad de México, México; ^3^ Laboratorio de Respiración Celular, Sección de Estudios de Posgrado e Investigación, Escuela Superior de Medicina, Instituto Politécnico Nacional, Ciudad de México, México; ^4^ Departamento de Química Orgánica, Escuela Nacional de Ciencias Biológicas, Instituto Politécnico Nacional, Ciudad de México, México; ^5^ Laboratorio de Modelado Molecular y Bioinformática y Diseño de Fármacos, Sección de Estudios de Posgrado e Investigación, Escuela Superior de Medicina, Instituto Politécnico Nacional, Ciudad de México, México

**This article has been corrected:** The correct author list is given below:

**Arturo Contis-Montes de Oca**^1,*^, Estefanía Rodarte-Valle^2,*^, Martha Cecilia Rosales-Hernández^**2**^**, Edgar Abarca-Rojano**^**3**^**, Saúl Rojas-Hernández**^**1**^**, Manuel Jonathan Fragoso-Vázquez**^**4**^**, Jessica Elena Mendieta-Wejebe**^**2**^**, Ana María Correa-Basurto**^**2**^**, Ismael Vázquez-Moctezuma**^**2**^ **and José Correa-Basurto**^**2,5**^

Original article: Oncotarget. 2018; 9:33368-33381. https://doi.org/10.18632/oncotarget.26077

